# Association between Telomere Length and Type 2 Diabetes Mellitus: A Meta-Analysis

**DOI:** 10.1371/journal.pone.0079993

**Published:** 2013-11-21

**Authors:** Jinzhao Zhao, Kun Miao, Haoran Wang, Hu Ding, Dao Wen Wang

**Affiliations:** 1 Institute of Hypertension and Department of Internal Medicine, Tongji Hospital, Tongji Medical College, Huazhong University of Science and Technology, Wuhan, China; 2 Genetic Diagnosis Center, Tongji Hospital, Tongji Medical College, Huazhong University of Science and Technology, Wuhan, China; Central China Normal University, China

## Abstract

**Background:**

Several epidemiological studies have examined the association between shortened telomere length and type 2 diabetes mellitus (T2DM), while the results remained conflicting. We conducted a meta-analysis to derive a more precise estimation of the relationship between them.

**Methods:**

We systematically reviewed the databases of PubMed, EMBASE, and Web of Science for all studies on the association between telomere length and T2DM. We conducted this study assessed by STATA 11.0. Data were summarized using random-effects or fixed-effects meta-analysis. The heterogeneity and publication bias among studies were examined by using χ^2^-based Q statistic test and Egger’s test, respectively.

**Results:**

Nine cohorts consisting of 5759 cases and 6518 controls were selected into the meta-analysis. The results indicated that shortened telomere length was significantly associated with T2DM risk (OR: 1.291; 95% CI: 1.112, 1.498; *P*<0.001) with heterogeneity (*I^2^ = *71.6%). When three cohorts responsible for the heterogeneity were excluded, the pooled OR for the remaining cohorts indicated a significant association between shortened telomere length and T2DM (OR: 1.117; 95% CI: 1.002, 1.246; *P* = 0.045) without heterogeneity.

**Conclusion:**

We found a statistically significant association between shortened telomere length and T2DM.

## Introduction

Telomeres are the DNA-protein structures capped at the ends of chromosomes in eukaryotic cells, which are very important for chromosome stability and integrity [1]. They shorten during each cycle of DNA replication and therefore have been implicated as a biomarker for cell aging [2]. It has been proved that shortened telomere length is associated with various age-related diseases, such as myocardial infarction [3], stroke [4], peripheral arterial disease (PAD) [5] and Alzheimer’s disease [6]. T2DM is one of the most common chronic diseases in the world. It has been proved to be an age-related disease, and therefore might be associated with telomere length as well. Jeanclos *et al* firstly reported the association between shortened telomere length and T2DM in 1998 [7], [8]. After the initial association discovery, several subsequent replication studies have been conducted in different population cohorts including Europeans, Asians and Africans [9], [10], [11], [12], [13], [14], [15]. However, the results remained conflicting. Most of the studies found that T2DM patients had shorter telomere length, while others reported a negative association between telomere length and T2DM [9], [12]. Considering that most of these studies have been conducted on relatively small numbers of subjects and no meta-analysis has specifically examined the relationship between shortened telomere length and T2DM, we conducted the meta-analysis expecting to give a more precise estimate of the relationship between telomere length and T2DM.

## Methods

### Search Strategy of Articles

We systematically searched MEDLINE, EMBASE, and Web of Science for all articles on the association between “diabetes” and “telomere” from their starting dates to Oct 31, 2012. We limited our search by English. In addition, hand searching was performed to identify potentially relevant studies.

### Selection Criteria and Exclusion Criteria

The literatures which meet the following criteria were selected into this meta-analysis: (1) case–control study or cohort study; (2) study aims at the association between telomere length and T2DM; and (3) study provides odds ratios (ORs) or sufficient information to estimate odds ratios (ORs) and their 95% confidence intervals (CIs).

The exclusion criteria were: (1) studies without sufficient information we need; (2) case subjects with multiple diseases; (3) case-only studies, review articles, commentaries, editorials, or case reports; (4) the studies have a sample size less than two hundred and haven’t given the number of cases and controls grouped by the median of the relative telomere length.

### Quality Assessment

The quality of studies was independently assessed by two authors (JZ and KM) using the following criteria: (1) Representativeness of cases, (2) Representativeness of controls, (3) Ascertainment of T2DM, (4) Ascertainment of controls, (5) Measuring telomere length, (6) Distribution of telomere length, (7) Association assessment (Table S1). Total scores ranged from 0 (worst) to 13 (best). Higher score represented a better quality.

### Data Extraction

Two reviewers (JZ and MK) extracted data from the selected articles independently. They compared the results and reached consistencies on all items. Characters extracted from the studies were as follows: journal, author, year of publication, cohort/study name, study type, geographic location of study, ethnicity of the study population, total number of cases and controls, average age at baseline, participants’ gender (male, female, combined), methods for telomere length measurement, the length of telomere and their standard error, unadjusted odds ratios (ORs) for individuals in the 50th percentile of shorter telomeres compared with those in the longer and the 95% CI of the ORs. Ethnic groups (categorized as Caucasian, Asian, or others) were extracted separately for studies consisting different racial descent subjects.

### Statistical Analysis

We performed the meta-analyses using the STATA 11.0 software (StataCorp, College Station, Texas, USA). We collected the unadjusted ORs for individuals in the 50th percentile of shorter telomeres compared with those in the longer and their 95% CI of the studies selected in the analysis. For the studies which did not provide ORs and 95% CI, we used the methods described by Wentzensen to compute the OR and 95% CI [16]. We assumed that the distribution of telomere length was normal, and divided the case and control subjects into two groups (the longer group and the shorter group) by using the mean telomere length of controls as a cut-point, and then we computed the probabilities among cases to fall into the shorter subgroup and longer subgroup, and after that we multiplied these probabilities by the total number of cases. We computed the ORs for the shorter compared with the longer group and their 95% CI using the χ^2^ test. With this method we converted the reported means and standard errors in cases and controls into ORs. However, studies with small sample size may be inappropriate to compute the OR in this way. So studies which didn’t provide the numbers of cases and controls grouped by the median of the relative telomere length and have a sample size less than two hundred were systematically excluded. Heterogeneity was evaluated by χ^2^-based Q statistic and *I^2^* statistic. The percentages of *I^2^* around 25%, 50%, and 75% indicate low, medium, and high heterogeneity, respectively [17]. The fixed-effects model was used to calculate the pooled OR when there was no significant heterogeneity (*P* more than 0.10 and *I^2^* less than 50%) among the included studies; otherwise, we used the random-effects model [18]. We conducted subgroup analyses according to ethnicity (Asian, Caucasian, and African), article quality (more than 8 and less than 8), sample size (more than 1000 and less than 1000), mean age (older than 60 and younger than 60), and gender (male, female and mixed). We performed meta-regression to find out the source of heterogeneity by fitting a co-variant (ethnicity, study quality, sample size, age and gender). Meanwhile, we also used the HETRED module of the STATA software to trace the responsible study for heterogeneity. Sensitivity analysis was performed to investigate the influence of each study on the overall result. In addition, publication bias was evaluated by funnel plot and Egger’s tests. All statistical testing was 2-sided and a *P*<0.05 was considered statistically significant.

## Results

### Literature Search

**Figure 1:**
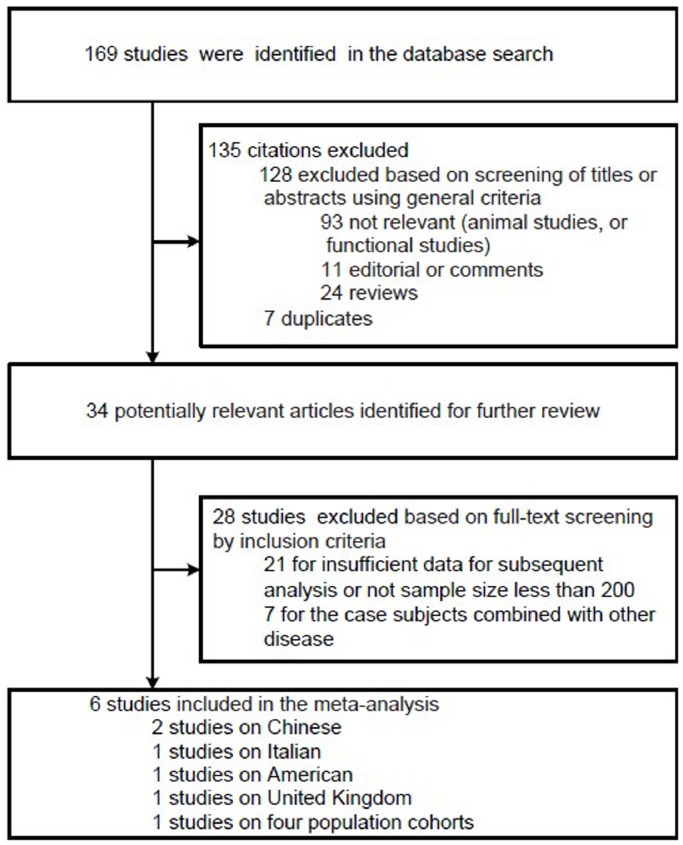
Flow chart for the process of selecting eligible publications.

On the initial search, we identified 169 studies by the keywords mentioned in the search strategy, of which 135 was excluded for they were not aiming at the association between telomere length and T2DM. After the abstracts or full texts were reviewed, 28 articles were excluded for the case subjects combined with other disease or without sufficient data for subsequent analysis. Finally, 6 studies from 5 nationalities consisting 9 population cohorts were selected into the analysis [9], [10], [11], [12], [13], [14] (Figure 1).The characteristics of studies included in the analysis were listed in Table 1. Among these articles, 2 studies reported results on Chinese [10], [11], 1 on Italian [12], 1 on American [13] and 1 on United Kingdom [14].

**Table 1:** Characteristics of studies examining the association between telomere length and T2DM included in the meta-analysis.

	Study	Year	Country	Design	Ethnicity	Case			Control			Quality assessment
						Total	Man	Age	Total	Man	Age	
1a	You *et al* 1 [9]	2012	USA	Prospective Study	Caucasian	1012	0	63.96	1023	0	63.92	7
1b	You *et al* 2 [9]	2012	USA	Prospective Study	African	400	0	60.97	819	0	60.95	7
1c	You *et al* 3 [9]	2012	USA	Prospective Study	Caucasian	162	0	60.15	331	0	60.21	7
1d	You *et al* 4 [9]	2012	USA	Prospective Study	Asian	101	0	63.37	207	0	63.58	7
2	Shen *et al* [10]	2012	Chinese	Case-control study	Asian	1936	30.2	64	2080	41.1	58	10
3	Xiao *et al* [11]	2011	Chinese	Case-control study	Asian	930	29.9	64.3	867	30.5	64.1	10
4	Testa *et al* [12]	2011	Italy	Cross-sectional study	Caucasian	217	59.7	65.6	400	55.6	65.1	9
5	Zee *et al* [13]	2009	USA	Case-control study	Caucasian	432	59.3	60	424	44.1	51	9
6	Salpea *et al* [14]	2009	U.K	Case-control study	Caucasian	569	59.4	70	367	100	53	9

### Results of Meta-analysis

**Figure 2:**
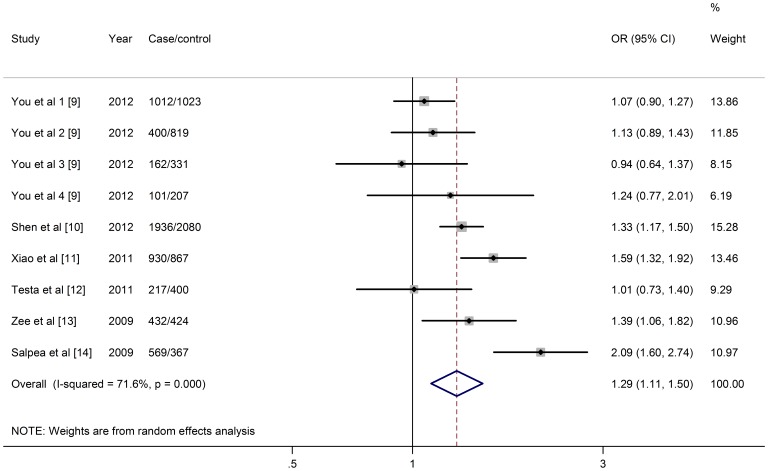
Meta-analysis of the association between telomere length and T2DM. Odds ratios (ORs) and 95% confidence intervals (CIs) for overall T2DM risk associated with relative telomere length (shorter vs. longer).

The raw data we extracted from the 9 population cohorts consists of 5759 cases and 6518 controls. When we pooled all eligible publications into the meta-analysis, significant association was found between shortened telomere length and T2DM (OR: 1.291; 95% CI: 1.112, 1.498; *P*<0.001) with heterogeneity (*I* ^2^ = 71.6%, χ^2^ = 28.13, *P*<0.001) (Figure 2). Subgroup analysis was further performed among these studies by ethnicity, study quality, sample size, age and gender (Table 2). The pooled ORs were increased in several subgroups, including: high quality studies, studies with younger control subjects and the mixed gender studies. High quality studies have an increased risk (OR: 1.452; 95% CI: 1.204, 1.753) compared with low quality studies (OR: 1.081; 95% CI: 0.952, 1.228). The control subjects of 3 reports (3647 control subjects) had an average age over 60, whereas other 7 cohorts (2871 control subjects) had an average age below 60. The pooled OR for younger subjects (OR: 1.543; 95% CI: 1.179, 2.018) was higher than the pooled OR for older (OR: 1.166; 95% CI: 0.975, 1.395). Meanwhile, the pooled OR for mixed gender studies (OR: 1.452; 95% CI: 1.204, 1.753) was higher than the pooled OR for studies focused on women (OR: 1.081; 95% CI: 0.952, 1.228).

**Table 2:** Subgroup analyses of the meta-analysis.

Variables	NO of cohorts	Sample		Shorter *vs*. longerOR (95% CI)	Q Statistic	*P* for Heterogeneity	*I*-squared Value	*P* Value Between Groups
		Case	Control					
**All**	9	5759	6518	1.291(1.112,1.498)	28.13	*P*<0.001	71.6%	NA
**HETRED** *	6	2317	3183	1.117(1.002,1.246)	4.07	0.539	0%	NA
**Ethnicity**								
Asian	3	2967	3154	1.409(1.230,1.615)	2.78	0.249	28.2%	
Caucasian	5	2392	2545	1.252(0.945,1.658)	21.45	*P*<0.001	81.4%	
Others	1	400	819	1.126(0.885,1.431)	0.00	NA	NA	*P* = 0.14
**Quality assessment**								
Over 8	5	4084	4138	1.452(1.204,1.753)	14.66	0.005	72.7%	
Under 8	4	1675	2380	1.081(0.952,1.228)	0.98	0.805	0.0%	*P*<0.001
**Sample size**								
More than 1000	4	4278	4789	1.270(1.075,1.500)	10.74	0.013	72.1%	
Less than 1000	5	1481	1729	1.299(0.958,1.762)	16.76	0.002	76.1%	*P* = 0.43
**Age**								
Over 60	6	2822	3647	1.166(0.975,1.395)	13.43	0.02	62.8%	
Under 60	3	2937	2871	1.543(1.179,2.018)	9.10	0.011	78.0%	*P* = 0.02
**Gender**								
Female	4	1675	2380	1.081(0.952,1.228)	0.98	0.805	0.0%	
Mixed	5	4084	4138	1.452(1.204,1.753)	14.66	0.005	72.7%	*P*<0.001

NA, not applicable;

* After excluding the key contributor studies to heterogeneity (Studies of Xiao *et al*, Shen *et al* and Salpea *et al*).

In the meta-regression analysis for heterogeneity, no participant characteristic (ethnicity, study quality, sample size, age and gender) was significantly correlated with the magnitude of genetic effect (all *P*>0.05). Next, we also used the HETRED module of the STATA software to find out the responsible study for heterogeneity. The studies of Xiao *et al*, Shen *et al* and Salpea *et al* were determined as the key contributors to the heterogeneity. After excluding these three studies, the overall heterogeneity decreased from 71.6% to 0% according to the *I^2^* assessment (Table 2). The pooled OR for the remaining six cohorts indicates a significant association between shortened telomere length and T2DM (OR: 1.117; 95% CI: 1.002, 1.246; *P* = 0.045).

### Sensitivity Analysis and Publication Bias

**Figure 3:**
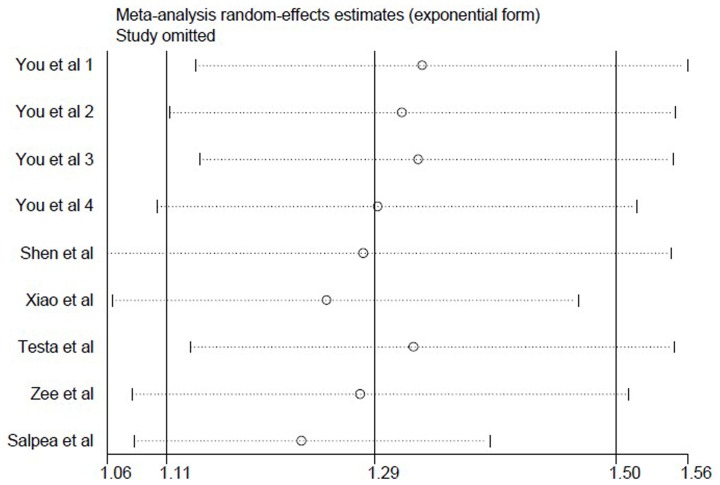
Sensitivity analyses of selected studies. Each line showed the recalculated pooled relative risk of remaining studies by omitting one study listed in the left volume a time.

**Figure 4:**
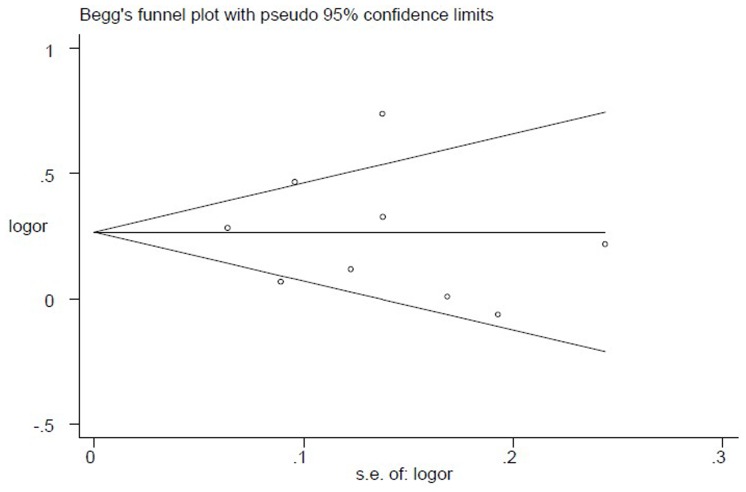
Begg’s funnel plot of selected studies. Asymmetry of the plot indicates publication bias. The horizontal line represents the meta-analysis summary estimate, the diagonal lines pseudo-95% CI limits about the effect estimate. logor, natural logarithm of the OR; s.e. of logor, standard error of logor.

Sensitivity analysis (exclusion of 1 study at a time) indicated that no single study changed the pooled ORs qualitatively (Figure 3), which suggested that the results of the meta-analysis were stable and reliable. Egger’s test suggested no publication bias in the current meta-analysis (*P* = 0.764) and the shape of the funnel plots seemed symmetrical (Figure 4). Thus publication bias might not have a significant influence on the result of this meta-analysis.

## Discussion

In this meta-analysis, we combined all together 9 cohorts consisting of 5759 T2DM cases and 6518 control subjects to evaluate the association between shortened telomere length and T2DM risk. The combined results showed that shortened telomere length is significantly associated with T2DM risk.

Previous studies have demonstrated that oxidative stress was a principal mechanism in the development and progression of T2DM [19], [20]. Elevated cellular glucose promotes the cellular oxidative stress [21]. Increased oxidative stress mediated cellular injury destroys the beta cells in the pancreas and decreases insulin sensitivity [22]. Furthermore, cellular injury and the decreased insulin sensitivity also promote the presence of cellular oxidative stress [23], [24]. In brief, oxidative stress was increased in T2DM patients. Clinical and experimental studies have indicated that oxidative stress elicits the deletion of telomeres [25], [26]. The G triplet of telomere is very sensitive to oxidative stress, and high-intensity stresses can cause telomere shorten by inducing telomeric double-strand breaks at high frequency [27], [28], [29]. So the telomere shortening in T2DM patients might be caused by increased oxidative stress and further studies are needed to elucidate the exact mechanisms underlying the association between telomere length and T2DM.

Although this meta-analysis showed significant association between shortened telomere length and T2DM, some results from subgroup analysis remind us of drawing the conclusion with caution. Firstly, previous studies have reported the variety of telomere between males and females [30]. Although the information is insufficient to compare gender difference in this analysis, the mixed gender studies had a higher OR than the studies focused on women subjects (1.452 vs. 1.081). Several hypotheses has explained the gender difference in telomere length and T2DM subjects, such as the higher stress exposure for males and the protective effect of estrogen in females. Secondly, age is another essential factor involved in telomere shortening [2]. All the studies have an average age between 60 and 70 in case subjects, so stratification analyses were conducted by the average age of control subjects (older than 60 or younger than 60). Both subgroups showed an association between telomere and T2DM, while the pooled OR for the studies with a younger control cohorts higher than the studies with an older control cohorts (1.543 vs. 1.166). It further identified that age has an influence on strength of association between telomere length and T2DM. Thirdly, several studies have reported the heterogeneity of telomere length within different population cohorts [9], [31], [32]. The results indicated that shortened telomere length was associated with T2DM in Asians, while not in Caucasians. The current study includes only one African cohort, and therefore the association in Africans should be evaluated in further studies with larger sample size.

Several limitations of the current meta-analysis should be noted. First, the publication bias might exist. In our meta-analysis, only studies indexed by the selected databases were included. Negative studies were less likely to be published in journals, resulting in potential overestimation of effect size. In addition, the exclusion of studies conducted in non-English speaking countries would have also introduced a considerable degree of bias. Second, telomere shortening is a cause or a consequence of diabetes remained to be clarified. Third, although all of the selected studies used the quantitative PCR based method to measure telomere length, the absolute telomere length was reported in different ways, including T/S ratio and KB, and therefore might provide us with less reliable evidence to make our conclusion. Fourth, seven of nine studies selected in the meta-analysis reported the adjusted OR. After adjusted for confounding variables (including BMI), their directions toward significant associations remain unchanged. While the detailed information on traditional risk factors was not available for analysis, the effects of those factors could not be adequately addressed in the present meta-analysis.

In summary, this meta-analysis provides evidence that shortened telomere length is associated with T2DM risk. Further prospective studies with larger sample size are needed to clarify whether telomere shortening is a cause or a consequence of diabetes.

## Supporting Information

Table S1 **Quality assessment of association studies between telomere length and T2DM.**
(DOC)

Checklist S1 **PRISMA checklist.**
(DOC)
