# Supplementary material for: Association between Telomere Length and Type 2 Diabetes Mellitus: A Meta-Analysis
Source: PLoS One. 2013 Nov 21;8(11):e79993. doi: 10.1371/journal.pone.0079993 (PMC3836967; doi:10.1371/journal.pone.0079993)
Supplement: Table S1 — Quality assessment of association studies between telomere length and T2DM. (DOC) [file pone.0079993.s001.doc]

| **Table S1:** **Scale for quality assessment of association studies between telomere length and T2DM** | |
| --- | --- |
| **Criteria** | **Score** |
| **Representativeness of cases** |  |
| Consecutive/randomly selected from case population with clearly defined sampling frame | **2** |
| Consecutive/randomly selected from case population without clearly defined sampling frame or with extensive inclusion/exclusion criteria | **1** |
| No method of selection described | **0** |
| **Representativeness of controls** | |
| Controls were consecutive/randomly drawn from the same sampling frame (ward/community) as cases | **2** |
| Controls were consecutive/randomly drawn from a different sampling frame as cases | **1** |
| Not described | **0** |
| **Ascertainment of cases** |  |
| Clearly described objective criteria for diagnosis of T2DM | **2** |
| Diagnosis of T2DM by patient self-report or by patient history | **1** |
| Not described | **0** |
| **Ascertainment of controls** |  |
| Controls were tested to screen out T2DM, i.e., measured OGTT | **2** |
| Controls were subjects who did not report T2DM; no objective testing | **1** |
| Not described | **0** |
| **Measuring telomere length** |  |
| Measuring under ‘‘blinded’’ condition | **1** |
| Unblinded or not mentioned | **0** |
| **Distribution of telomere length** |  |
| Strict normal distribution | **2** |
| Abnormal distribution with log- transformed or log_e_-transformed | **1** |
| No checking for distribution | **0** |
| **Association assessment** |  |
| Assess association between telomere length and T2DM with appropriate statistics and adjustment for confounders | **2** |
| Assess association between telomere length and T2DM with appropriate statistics without adjustment for confounders | **1** |
| Inappropriate statistics used | **0** |
